# Consistent effects of the genetics of happiness across the lifespan and ancestries in multiple cohorts

**DOI:** 10.1038/s41598-023-43193-9

**Published:** 2023-10-12

**Authors:** Joey Ward, Laura M. Lyall, Breda Cullen, Rona J. Strawbridge, Xingxing Zhu, Ioana Stanciu, Alisha Aman, Claire L. Niedzwiedz, Jana Anderson, Mark E. S. Bailey, Donald M. Lyall, Jill P. Pell

**Affiliations:** 1 School of Health and Wellbeing, University of Glasgow, 1 Lilybank Gardens, Glasgow, G12 8RZ UK; 2 Cardiovascular Medicine Unit, Department of Medicine Solna, Karolinska Institutet, Stockholm, Sweden; 3 Health Data Research UK, Glasgow, UK; 4 School of Life Sciences, University of Glasgow, Glasgow, Scotland, UK

**Keywords:** Genetic association study, Genomics, Human behaviour, Emotion

## Abstract

Happiness is a fundamental human affective trait, but its biological basis is not well understood. Using a novel approach, we construct LDpred-inf polygenic scores of a general happiness measure in 2 cohorts: the Adolescent Brain Cognitive Development (ABCD) cohort (N = 15,924, age range 9.23–11.8 years), the Add Health cohort (N = 9129, age range 24.5–34.7) to determine associations with several well-being and happiness measures. Additionally, we investigated associations between genetic scores for happiness and brain structure in ABCD (N = 9626, age range (8.9–11) and UK Biobank (N = 16,957, age range 45–83). We detected significant (p.FDR < 0.05) associations between higher genetic scores vs. several well-being measures (best r^2^ = 0.019) in children of multiple ancestries in ABCD and small yet significant correlations with a happiness measure in European participants in Add Health (r^2^ = 0.004). Additionally, we show significant associations between lower genetic scores for happiness with smaller structural brain phenotypes in a white British subsample of UK Biobank and a white sub-sample group of ABCD. We demonstrate that the genetic basis for general happiness level appears to have a consistent effect on happiness and wellbeing measures throughout the lifespan, across multiple ancestral backgrounds, and multiple brain structures.

## Introduction

Happiness is the core positive emotional state. As a trait that is affected in clinical outcomes (e.g. lack of happiness in depression) it is a positive valence Research Domain Criteria (RDOC) trait^1^. At the genetic level it is more often analysed as part of a wider concept of well-being^2,3^. There is evidence that, generally, an individual has a baseline happiness level which remains relatively stable over time^4^ even after major positive or negative life events such as winning the lottery or being the victim of an accident^5^.

To date, the largest genome-wide association study (GWAS) was performed in UK Biobank by Baselmans and Bartels^6^ (N ~ 222k) and is comprised of a meta-analysis of the question “*In general, how happy are you?*” which has been asked twice longitudinally (5 years apart approximately). A GWAS was performed on each instance of the question and the results combined in a meta-analysis. The impact of this genetic liability to higher or lower happiness has little understanding, including key aspects such as influence over the structural brain substrates.

Here we report the use of happiness polygenic scores (PGS) using the output of Baselmans and Bartels^6^ in three cohorts across multiple age demographics: the Adolescent Brain Cognitive Development (ABCD) cohort^7^ (children aged 9–11) and the National Longitudinal Study of Adolescent to Adult Health (Add Health)^8^ (adults aged 25–35) and the UK Biobank (middle-to-older age; 40–70). This study aimed to determine: (1) whether increased genetic loading of this happiness measure is significantly associated with happiness and well-being measures in independent cohorts that span differing age ranges and different ancestries compared to the discovery GWAS cohort; and (2) to investigate whether genetic predisposition for general happiness level is associated with average differences in key aspects of brain structure and white matter integrity measures with previous evidence of relating to psychological health.

## Results

### Happiness and well-being measures

#### ABCD

In the white subsample, genetic loading for happiness associated with the combined wellbeing measures (β = 0.14, S.E. = 0.04, p.FDR = 0.02), and five out of the nine well-being measures after correcting for multiple testing (Table S1). Reporting being delighted (β = 0.021, S.E. = 0.008, p.FDR = 0.016), calm (β = 0.029, S.E. = 0.007, p.FDR = 7.4 × 10^–5^), enthusiastic (β = 0.038, S.E. = 0.008, p.FDR = 5.8 × 10^–6^), confident (β = 0.034, S.E. = 0.008, p.FDR = 5.8 × 10^–6^) and energetic (β = 0.02, S.E. = 0.007, p = 0.008) all showed positive associations. Full results in the other ancestries can be found in Table S1.

**Figure 1:**
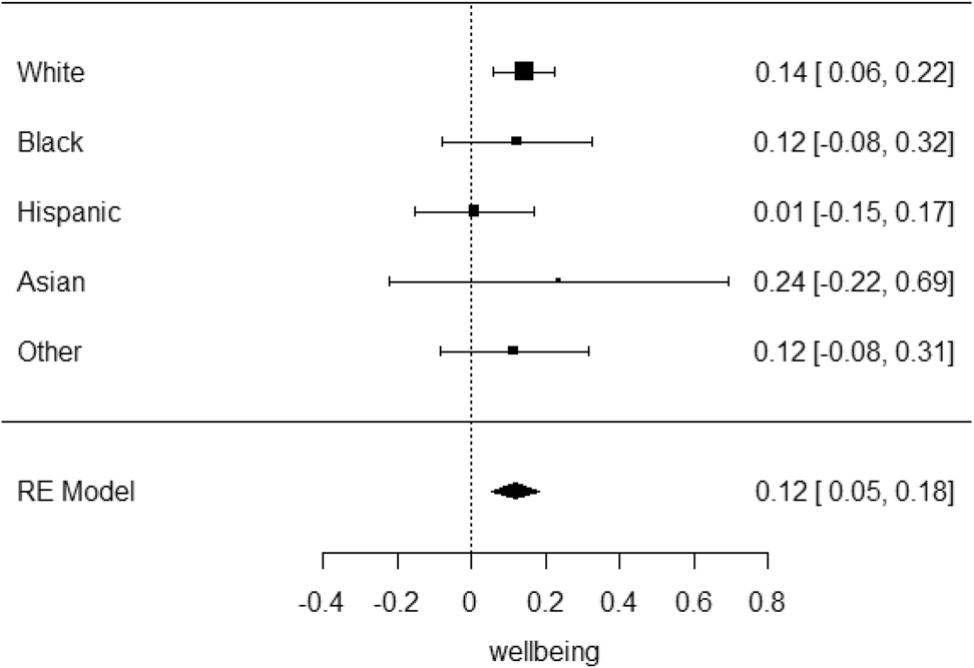
Forest plot of the random effects meta-analysis of wellbeing score in the ABCD cohort.

In the meta-analysis of the whole sample, the combined wellbeing measures (β = 0.12, S.E. = 0.03, p.FDR = 0.004, Fig. 1) and three of the nine well-being measures associated with genetic loading for happiness after correcting for multiple testing (Table 1, Figure S1). Being, confident (β = 0.022, S.E. = 0.009, p.FDR = 0.00495), energetic (β = 0.02, S.E. = 0.009, p.FDR = 0.0495) and able to concentrate (β = 0.027, S.E. = 0.01, p.FDR = 0.0495). As with the white subsample, all showed a positive association with genetic loading for happiness. Heterogeneity I^2^ was moderate (~ 50) in the significant individual item wellbeing measures but low (0) in the overall wellbeing measure (Table 1).

**Table 1:** Association of happiness PGS with wellbeing measurement-analysis in the ABCD cohort.

Trait	Beta	S.E	p	I^2^	N	p.FDR
Wellbeing	0.117	0.033	0.000397	0	15,402	0.00397
Attentive	− 0.0131	0.00718	0.0677	0	15,568	0.1354
Delighted	0.00805	0.0109	0.459	44.6	15,577	0.51
Calm	0.00743	0.00988	0.452	58.5	15,599	0.51
Relaxed	0.0178	0.0109	0.102	50.9	15,561	0.17
Enthusiastic	0.0198	0.0146	0.176	70.5	15,589	0.25
Interested	− 0.00515	0.0113	0.65	51.9	15,575	0.65
Confident	0.0217	0.00933	0.0198	51	15,571	0.0495
Energetic	0.0229	0.00975	0.019	45.2	15,575	0.0495
Concentrate	0.0266	0.0103	0.00982	55.5	15,567	0.0491

#### Add health

**Figure 2:**
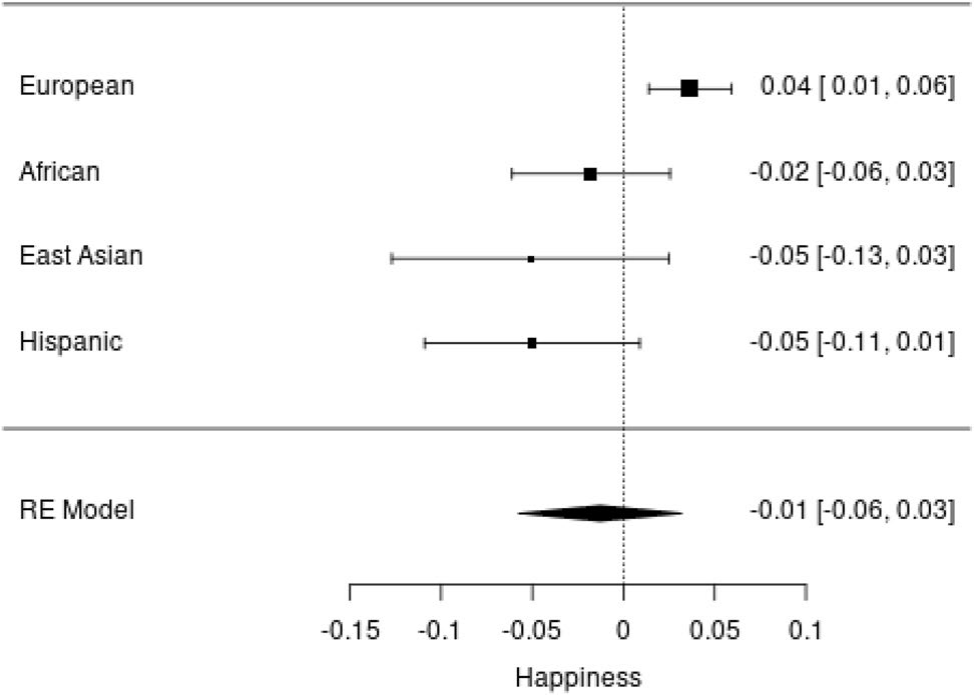
Forest plot of the random effects meta-analysis of happiness measure in the Add Health cohort.

A significant association was found in the LDpred analysis in the European ancestry subsample (β = 0.04, S.E. = 0.01, P = 0.002, Table S2) but we did not detected a significant association in the meta-analysis of whole sample (β = − 0.019, S.E. = 0.01, P = 0.57, Fig. 2). We note that heterogeneity was high (I^2^ = 73.6).

### Brain structure

#### UK Biobank

After correcting for multiple testing via FDR there were no brain phenotype measures that showed significant association with genetic loading for happiness (Table S3). Several regions were nominally significant. With greater genetic loading for happiness was correlated with larger volumes: white matter volume (β = 0.020, S.E. = 0.007, P = 0.005), the right accumbens (β = 0.020, S.E. = 0.007, P = 0.006), the left hippocampus tail (β = 0.016, S.E. = 0.007, P = 0.016) and grey matter volume (β = 0.011, S.E. = 0.007, P = 0.046). Both the left and right thalamus were borderline significant before FDR correction (left: β = 0.013, S.E. = 0.007, P = 0.053, right: β = 0.013, S.E. = 0.007, P = 0.05).

The WM integrity measure gMD showed a nominally significant negative association (β = − 0.014, S.E. = 0.007, P = 0.048) while gFA (β = 0.011, S.E. = 0.008, P = 0.15) showed a positive correlation (i.e. both were protective) however gFA was not significant.

#### ABCD

In the white subsample, the volume of: the left pallidum (β = 0.055, S.E. = 0.015, p.FDR = 0.002), right cerebral white matter (β = 0.052, S.E. = 0.014, p.FDR = 0.002), total white matter (β = 0.05, S.E. = 0.014, P.FDR = 0.002), left cerebral white matter (β = 0.05, S.E. = 0.014, p.FDR = 0.002), the left thalamus proper (β = 0.047, S.E. = 0.014, p.FDR = 0.002), the right thalamus proper (β = 0.045, S.E. = 0.014, p.FDR = 0.006), total grey matter(β = 0.043, S.E. = 0.014, p.FDR = 0.006), the total frontal lobe (β = 0.04, S.E. = 0.015, p.FDR = 0.01), the right hippocampus (β = 0.037, S.E. = 0.015, p.FDR = 0.03) were significant after FDR correction. The right caudate (β = 0.032, S.E. = 0.015, P = 0.037) was only significant before FDR correction. All significant regions showed a positive correlation with genetic loading for happiness (Table S4).

For brain integrity measures in the white subsample, gMD showed a negative association while gFA showed a positive correlation (i.e. both protective), however neither association was statistically significant (Table S4).

In the meta-analysis of the whole sample, no brain regions were significantly associated with the happiness PGS after FDR correction. However, the volume of: the right amygdala (β = 0.03, S.E. = 0.01, P = 0.009, p.FDR = 0.19), and the right putamen (β = 0.025, S.E. = 0.01, P = 0.02, p.FDR = 0.21), were nominally associated (Table S5).

For brain integrity measures in the whole sample, gFA showed positive association while gMD showed a negative correlation, but these were not significant (Table S5).

## Discussion

The results of the LDpred PGS analyses in ABCD and Add Health showed that genetic liability toward happiness has fairly consistent effects across the lifespan from age 12 to 73 including across multiple ancestral backgrounds with the exception of the non-white ancestries of Add Health. The results of these models for the ABCD cohort show that genetic loading for happiness may drive certain aspects of an individual’s overall wellbeing (e.g., being calm, confident, energetic, able to concentrate), as well as aspects of brain structure known to underlie psychological as well as cognitive health.

### Imaging

The results of the MRI analyses in the UK Biobank returned only nominally significant regions that were associated with genetic loading for happiness and this may be an underestimate due to the known biases particularly within the MRI subsample—a bias evidenced to meaningfully underestimate effect estimates^9^. We detected several brain regions associated with genetic loading for happiness in the ABCD cohort, which is more representative of the populations from which it is drawn and these models were appropriately weighted.

Several brain regions have already been identified in hedonic brain circuitry such as the ventral pallidum and nucleus accumbens^10^. Models by Loonen et al.^11^ suggest that many of the regions identified in our analyses regulate both pleasure and happiness pathways. They suggest the caudate nucleus, putamen and core of the accumbens nucleus are involved in experiencing pleasure and the amygdala, bed nucleus of the stria terminalis and accumbens nucleus are involved in experiencing happiness.

We identified associations with volumes of the left ventral pallidum in the white subsample of ABCD. We did not find any significant association with the nucleus accumbens in either ABCD analysis, but it was nominally significant in the UK Biobank model. We also note a consistent direction of effect of both these regions across the ABCD and UK Biobank analyses.

The hippocampal regions have been shown to be involved in hedonic neural circuitry for example in the meta-analysis of Tanzer and Wayandt^12^. Our results reflect these findings in the white sub-sample of the ABCD cohort but only in the right hippocampus.. The left hippocampus tail was nominally significant in the UK Biobank analysis but all other tested hippocampal regions and subregions showed no association.

The frontal lobe has been implicated in hedonic emotions^10,13^ and we showed that greater frontal lobe volume significantly correlated with genetic loading for happiness in the white subsample of ABCD, again we also note a consistent direction of effect with the frontal lobe general factor used in the UK Biobank analysis. We also detected a positive correlation with grey matter volume in the ABCD analyses and a nominally significant association with same direction of effect reflected in the UK Biobank cohort, an association that has been identified elsewhere^14^. We also identify a novel finding of greater white matter volume in ABCD which is reinforced by the consistent direction of effect in UK Biobank.

## Strengths and limitations

UK Biobank is not representative of the UK general population in that participants are generally healthier and have a higher socioeconomic status than the general population and therefore may have a different happiness level distribution than the UK as a whole^15^. The MRI sample is even less representative in that participation was slightly biased towards the fitter, healthier participants in UK Biobank ^9^.

A similar issue arises in the PGS analyses in that those of European ancestry were used to establish LD structure of the genome. This was due to the lower numbers of non-European ancestry participants in these cohorts. As a result, our findings possibly underestimate the effect in non-European ancestries.

## Conclusions

While previous studies demonstrated a genetic underpinning to stable happiness, there is a lack of understanding regarding the impact of such liability on wellbeing and aspects of brain structure. These analyses demonstrate that general happiness level has a genetic contribution which has a consistent effect across age groups and ancestral backgrounds, as well as associations with brain structure regardless of current happiness state. These analyses not only help increase our understanding of psychology and neurodevelopment, the novel methodology of using UK Biobank participants as a reference panel for LDpred PGS could be adapted for a wide range of other phenotypes. Even still, it would be of benefit to perform further analyses using larger datasets with less bias towards those of European ancestry.

## Methods

### Cohorts, genotyping and phenotyping

#### Adolescent brain cognitive development (ABCD)

##### ABCD cohort description

The Adolescent Brain Cognitive Development (ABCD) cohort is a longitudinal study of brain development and child health^7^. Investigators at 21 sites around the USA conducted repeated assessments of brain maturation in the context of social, emotional, and cognitive development, as well as a variety of health and environmental outcomes. We analysed data from release 3.0. At the time of the survey questions, the children ranged in age from 9 to 12 years. Informed written consent was provided by parents and assent was provided by children. The ABCD research protocol approved was approved by the Institutional Review Board of University of California San Diego (IRB# 160091)^16^.

Data used in the preparation of this article were obtained from the Adolescent Brain Cognitive DevelopmentSM (ABCD) Study (https://abcdstudy.org), held in the NIMH Data Archive (NDA). This is a multisite, longitudinal study designed to recruit more than 10,000 children age 9–10 and follow them over 10 years into early adulthood. The ABCD Study^®^ is supported by the National Institutes of Health and additional federal partners under award numbers U01DA041048, U01DA050989, U01DA051016, U01DA041022, U01DA051018, U01DA051037, U01DA050987, U01DA041174, U01DA041106, U01DA041117, U01DA041028, U01DA041134, U01DA050988, U01DA051039, U01DA041156, U01DA041025, U01DA041120, U01DA051038, U01DA041148, U01DA041093, U01DA041089, U24DA041123, U24DA041147. A full list of supporters is available at https://abcdstudy.org/federal-partners.html. A listing of participating sites and a complete listing of the study investigators can be found at https://abcdstudy.org/consortium_members/. ABCD consortium investigators designed and implemented the study and/or provided data but did not necessarily participate in the analysis or writing of this report. This manuscript reflects the views of the authors and may not reflect the opinions or views of the NIH or ABCD consortium investigators.

The ABCD data repository grows and changes over time. The ABCD data used in this report came from https://doi.org/10.15154/1526432) DOIs can be found at https://dx.doi.org/10.15154/1526432. All methods were carried out in accordance with relevant guidelines and regulations.

##### ABCD genotyping

DNA was extracted from saliva samples of the ABCD participants^17^. These samples were genotyped on the Affymetrix NIDA SmokeScreen Array (Affymetrix, Santa Clara, CA, USA). The QC procedures are described in full at the following URL: https://doi.org/10.15154/1503209.

ABCD genetic principal components (GPCs) were created using genotyped only SNPs using plink-pca flag.

##### ABCD phenotyping and exclusion criteria

A set of questions taken from the ABCD Youth NIH Toolbox Positive Affect Items was used. These questions measured aspects of positive emotions and affective well-being in the past week, specifically being attentive, delighted, calm, relaxed, enthusiastic, interested, confident, energetic and able to concentrate. Responses were measured as ‘not true’, ‘somewhat true’ or ‘very true’. Each item was analysed separately as well as a combined score that was the sum of responses to the individual questions. In addition to the happiness PGS the models were adjusted for age, sex, and principal genetic components (PGCs) 1–8.

As the initial UK Biobank GWAS was run in the white British sub-group, testing was performed firstly in the white (as defined by ABCD) participants and secondly in the whole sample, with ancestry treated as a factor variable. The other ancestral backgrounds of this cohort as defined by ABCD are; White, Black, Hispanic, Asian, and Other (Table S6).

#### ABCD MRI brain scans

Creation of the derived MRI variables from the ABCD cohort has been described in detail elsewhere^18^. For the purposes of this study, total frontal lobe volume was derived by summing the 22 frontal lobe subsection variables of the left and right hemisphere^19^. Additionally, we looked at total grey and white matter volume and left and right hippocampus volume. The hippocampal body and tail regions and white matter hyperintensity volume were not available for replication. All outcomes were transformed into z scores and all models were adjusted for the happiness PGS, age, sex, PGCs 1–8, and MRI site. For models that included participants from different ancestries, a factor variable for ancestry was included (Table S7). Models were weighted to match the American community survey (ACS) data by the weighting variable “acs raked propensity score”. Relationship filtering was also performed removing one individual at random from any pair of participants with valid phenotypes, who were determined to be related by ABCD.

#### Add health

##### Add health cohort description

Add Health is a nationally representative cohort study of more than 20,000 adolescents from the USA who were aged 12–19 years at baseline assessment in 1994–95. They have been followed through adolescence and into adulthood with five in-home interviews in five waves (I–V) conducted in 1995, 1996, 2001–2002, 2008–2009 and 2016–2018. In this analysis, participants ranged from 24.3 to 34.7 years old, 53% were female and 62% were non-Hispanic white. The study was approved by the University of California San Diego Institutional Review Board (IRB #190002XX). Informed consent was obtained from all subjects.

##### Add health genotyping

Saliva samples were obtained as part of the Wave IV data collection. Two Illumina arrays were used for genotyping, with approximately 80% of the sample genotyped with the Illumina Omni1-Quad BeadChip and the remainder of the group genotyped with the Illumina Omni2.5-Quad BeadChip. After quality control, genotyped data were available for 9974 individuals (7917 from the Omni1 chip and 2057 from the Omni2 chip) on 609,130 SNPs present on both genotyping arrays^20^. Imputation was performed separately for European ancestry (imputed using the HRC reference panel) and non-European ancestry samples (imputed using the 1000 Genomes Phase 3 reference panel)^21^. For more information on the genotyping and quality control procedures see the Add Health GWAS QC report online at: https://addhealth.cpc.unc.edu/wp-content/uploads/docs/user_guides/AH_GWAS_QC.pdf.

Add Health Genetic Principal components (variable name pspcN, where N is the number of the PC) were derived centrally by Add Health. To prevent identification of individuals they are randomly reordered in sets of 5, i.e. PCs 1–5 were reordered so PC1 was may not be the PC with the largest variance. We adjusted models for the first 2 sets of PCs i.e. GPCs 1–10.

##### Add health phenotyping and exclusion criteria

The outcome happiness variable was collected during the at-home interview of Wave IV and was derived from the response to the question: “How often was the following true during the past seven days? You felt happy.” Responses were given as: “never or rarely”; “sometimes”; “a lot of the time”; “most of the time or all of the time”; “refused”; “don't know”. Those who responded with the latter two options were excluded. Remaining categories were coded from “never” = 0 to “all of the time” = 3.

Ancestry in Add Health is defined in the ‘psancest’ variable as European, African, Hispanic and East Asian (Table S8). Additionally, Add Health provides a weighting variable to make the results reflective of the US population. In these analyses the models were weighted by the Wave IV variable ‘gswgt4_2’.

#### UK Biobank

##### UK Biobank cohort description

UK Biobank is a cohort of over half a million UK residents, aged from approximately 40–70 years at baseline. It was created to study environmental, lifestyle and genetic factors in middle and older age^22^. Baseline assessments occurred over a 4-year period, from 2006 to 2010, across 22 UK centres. These assessments were comprehensive and included social, cognitive, lifestyle and physical health measures.

UK Biobank obtained informed consent from all participants, and this study was conducted under generic approval from the NHS National Research Ethics Service (approval letter dated 29 June 2021, Ref 21/NW/0157) and under UK Biobank approvals for application #71392 ‘Investigating complex relationships between genetics, exposures, biomarkers, endophenotypes and cardiometabolic, inflammatory, immune and brain-related health outcomes’ (PI Rona Strawbridge; GWAS)#17689 (PI Donald Lyall; imaging).

##### UK Biobank genotyping

In March 2018, UK Biobank released genetic data for 487,409 individuals, genotyped using the Affymetrix UK BiLEVE Axiom or the Affymetrix UK Biobank Axiom arrays (Santa Clara, CA, USA) containing over 95% common content. Pre-imputation quality control, imputation and post-imputation cleaning were conducted centrally by UK Biobank (described in the UK Biobank release documentation)^23^.

#### UK Biobank phenotyping and exclusion criteria

##### UK Biobank MRI brain scans

Several structural and functional brain MRI measures are available in UK Biobank as imaging derived phenotypes (IDPs)^24^. The brain imaging data, as of January 2021, were used (N = 47,920). Participants were excluded if they had responded to either of the happiness questions used for the GWAS meta-analysis, were missing more than 10% of their genetic data, if their self-reported sex did not match their genetic sex, if they were determined by UK Biobank to be heterozygosity outliers, and if they were not of white British ancestry (classified by UK Biobank based on self-report and genetic principal components)^23^.

Brain imaging data used here were processed and quality-checked by UK Biobank and we made use of the IDPs^25,26^. Details of the UK Biobank imaging acquisition and processing, including structural segmentation and white matter diffusion processing, are freely available from three sources: the UK Biobank protocol: http://biobank.ctsu.ox.ac.uk/crystal/refer.cgi?id=2367 and documentation: http://biobank.ctsu.ox.ac.uk/crystal/refer.cgi?id=1977 and in protocol publications (https://biobank.ctsu.ox.ac.uk/crystal/docs/brain_mri.pdf).

We investigated key imaging substrates previously associated with psychological health e.g., mood disorder, cognitive health. Total white matter hyperintensity volumes were calculated on the basis of T1 and T2 fluid-attenuated inversion recovery, derived by UK Biobank. White matter hyperintensity volumes were log-transformed due to a positively skewed distribution. We constructed general factors of white matter tract integrity using principal component analysis. The two separate unrotated factors used were fractional anisotropy (FA), gFA, and mean diffusivity (MD), gMD, previously shown to explain 54% and 58% of variance, respectively^27^. We constructed a general factor of frontal lobe grey matter volume using 16 subregional volumes as per Ferguson et al.^27^. Total grey matter and white matter volumes were corrected for skull size (by UK Biobank). Models were adjusted for the happiness PGS, age, sex, PGCs 1–8.

### Analyses

#### LDpred genetic score generation

##### UK Biobank

LDpred^28^ established the LD structure of the genome using a reference panel of 1000 unrelated white British UK Biobank participants (the PGS training set). These participants had not been used in the discovery GWAS or have valid MRI data and passed the same QC as described above. SNPs were excluded if they had MAF < 0.01, had HWE P < 1 × 10^–6^ or had imputation score < 0.8. Scores were then created in the validation set using an infinitesimal model. Models using polygenic scores (PGS) derived using LDpred were adjusted for age, sex, genotyping array and the first eight GPCs.

##### ABCD and Add Health

Due to the lower cohort size of ABCD and Add Health, it would not have been possible to remove 1000 participants from the analyses to use as a training set without markedly reducing the power of the analyses. Therefore, we used the same 1000 unrelated UK Biobank participants as the training set to establish LD and this was used to generate the PGS for the participants in these datasets^29^. The only additional step was to find the SNPs that were found in both the training (UK Biobank) and validation (ABCD and Add Health) datasets and passed the same SNP filtering criteria in both datasets, with an additional filter that MAF threshold was set at > 0.01^30^. The number of SNPs in each LDpred PGS can be found in supplementary table (S9).

For each pair of related individuals (as determined by ABCD using variables genetic paired subjected 1–4) one participant was excluded at random. Models were adjusted for age at interview, sex and the first 10 GPCs. For multi-ancestry models, ancestry was treated as a factor variable.

p values for analyses were false discovery rate (FDR)-adjusted^31^.

### Supplementary Information

Supplementary Information.

Supplementary Figure 1.

Supplementary Table 1.

Supplementary Table 2.

Supplementary Table 3.

Supplementary Table 4.

Supplementary Table 5.

Supplementary Table 6.

Supplementary Table 7.

Supplementary Table 8.

Supplementary Table 9.

## Data Availability

The phenotype and genotype data used for these analyses are available from their respective websites: UK Biobank: https://www.ukbiobank.ac.uk/enable-your-research/apply-for-access; Add Health: https://addhealth.cpc.unc.edu/data/; ABCD: https://nda.nih.gov/abcd/request-access.html; GWAS summary statistics were obtained via email from the GWAS author.
