# Supplementary figures and images for: Consistent effects of the genetics of happiness across the lifespan and ancestries in multiple cohorts
Source: Sci Rep. 2023 Oct 12;13:17262. doi: 10.1038/s41598-023-43193-9 (PMC10570373; doi:10.1038/s41598-023-43193-9)

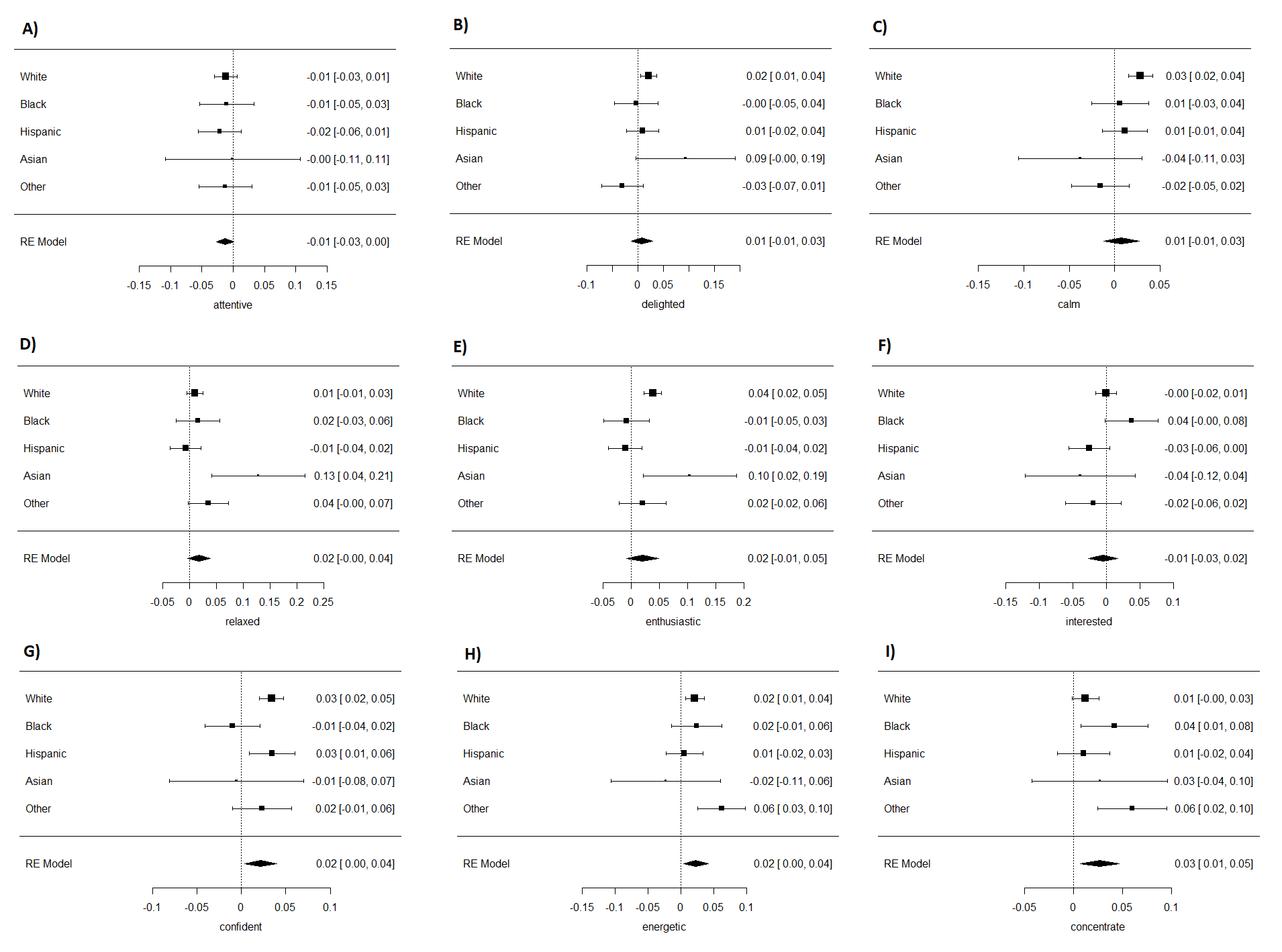

Supplement: Supplementary file 2 — Supplementary Figure 1. [file 41598_2023_43193_MOESM2_ESM.png]
